# Social capital and COVID-19 vaccination in prisons: a survey of attitudes, beliefs, and motivations

**DOI:** 10.1093/pubmed/fdaf033

**Published:** 2025-03-30

**Authors:** Stephanie E Perrett, Daniel Rhys Thomas, Christopher W N Saville

**Affiliations:** Communicable Disease Inclusion Health Programme, Public Health Wales, Floor 4 Number 2 Capital Quarter, Tyndall Street, Cardiff CF10 4BZ; Communicable Disease Surveillance Centre, Public Health Wales, Health Protection Team, Floor 4, Number 2 Capital Quarter, Tyndall Street, Cardiff, CF10 4BZ; School of Psychology and Sports Science, Bangor University, Brigantia Building, Penrallt Road, Bangor, Gwynedd, LL57 2AS

**Keywords:** COVID-19, prison health, social capital, social determinants of health, trust, vaccine

## Abstract

**Background:**

UK prisons were profoundly affected by the COVID-19 pandemic. Despite this, uptake of the COVID-19 vaccination remained low in these settings. Social roles, relationships, and freedoms influence vaccine uptake. Prisons are isolated by design and may foster mistrust that negatively influences vaccine uptake. Prisons are also unique communities with their own social networks and relationships.

**Methods:**

We undertook a questionnaire survey across all six prisons in Wales, UK, to gather data on vaccine behaviour, attitudes, and other influencing factors. We fitted binomial generalised linear mixed effects models to identify predictors of vaccination.

**Results:**

Surveys were completed by 727 prison residents. We found low vaccination uptake in younger cohorts, those serving short sentences, and those who perceived themselves to be in poorer health. Those reporting low levels of trust towards others and those reporting fewer sources of social support in prison were less likely to be vaccinated.

**Conclusion:**

Our findings suggest that building a prosocial atmosphere in prison and strengthening relationships and trust between prison staff and residents would positively influence vaccine uptake. Specific message framing should be considered to address the beliefs and motivations most prevalent in this population group, rather than focussing simply on enhancing the opportunity for vaccination.

## Introduction

UK prisons were profoundly affected by the COVID-19 pandemic, with over 30 000 confirmed cases and 159 deaths from suspected or confirmed COVID-19.^1^ Rates of COVID-19 in UK prisons appeared higher than in the general community, with the first wave of the pandemic seeing 7.6 confirmed cases per 1000 people in prison compared to 4.9 outside.^2^ Prisons hold populations with high health needs in crowded conditions, posing challenges for infection control.^3^^,^^4^ Interventions have been proposed for outbreak management in prisons, including improved hygiene, cleaning, mask-wearing, ventilation, decarceration, and cohorting,^5–7^ but vaccination of prison residents remains a public health priority to reduce morbidity and mortality and limit the spread of infection.^8^ Vaccine uptake in prison residents appears lower than in community counterparts globally.^9^^,^^10^ There is limited evidence of approaches to promote vaccination in prisons.^11^

COVID-19 vaccination uptake has been linked to social capital^12^^,^^13^—a multifaceted concept, comprising trust, social connectedness, and norms of reciprocity, all measurable components.^14^ Given the breadth of the construct, and how distinct facets can differentially influence health,^15^ it is important to be clear about which aspects of social capital are being measured in a given setting.^16^ We view prison as a distinct social network with relationships that manifest differently to the wider community. Social organisation in prisons can be influenced by gender, ethnicity, age, criminal experience, geography, gang affiliations, and relationships with staff. Social hierarchies may have violent offenders at the top and those who are considered weak or ostracised at the bottom.^17–19^ Prisons isolate by design, challenging bridging (network building) social capital. Prisons can foster deep mistrust,^17^^,^^20^ threatening bonding social capital (trust and reciprocity) that may undermine health decisions. Whilst social capital in prisons is poorly understood, evidence suggests that it can increase participation in health interventions.^21^^,^^22^ Social capital, through the role of peer support, has been shown to positively influence hepatitis C treatment adherence in prison.^23^ Those in prison who are socially bonded by race, ethnicity, religion, or exercise have been shown to have better health outcomes.^22^ Research is needed to understand the role of social capital in vaccination decisions. We used a survey design to measure the impact of relationships and trust within the prison on the decision to accept COVID-19 vaccination.

## Methods

### Ethics and permissions

The study was approved by NHS REC (22/WA/0187) and HMPPS National Research Committee (2022-196). Due to the sensitivity of the data, we regret that we do not have ethical approval to share data.

### Setting and fieldwork

There are 5300 residents in six male prisons in Wales.^13^ There are no female prisons in Wales. Prisons in England and Wales are governed by the UK Ministry of Justice, with primary healthcare services provided by the National Health Service and social care provided or commissioned by local authorities.^24^ Prison residents are entitled to the same vaccines as those in the community.

Between October and December 2022, ~1500 paper questionnaires (enough for 30% of the prison population at each site) were distributed in all six prisons in Wales. The sample size was based on the experience of previous surveys run in these prisons. Participation was voluntary, and responses remained confidential. Participants needed to be 18 years old or over and able to communicate in Welsh or English. Questionnaire distribution was led by nominated custodial staff and prisoner peer representatives who underwent training from the research team. Leaflets describing the survey were distributed across all prison wings by the nominated staff. Peer representatives were available to answer questions about the research and support participation. Participants completed consent forms alongside the questionnaire. Responses were sealed and sent to a research company, Beaufort Research, for collation. Prisons were blinded, identified by a code known only to the chief investigator.

### Measures

The questionnaire was based on a community survey on vaccination attitudes, behaviour, and social capital in Wales.^25^ Content was adapted to ensure appropriateness for a prison population. The full questionnaire is available at https://osf.io/cr4bz/files/osfstorage.

Variables were grouped into three categories: sociodemographic characteristics, vaccination attitudes and behaviour, and social capital and support.

Sociodemographic variables included age, ethnicity, and education. Health-related characteristics included self-reported general health, whether the respondent had been vaccinated against influenza, and whether the respondent had been vaccinated against hepatitis-B.

Sentence-related characteristics were included as a sociodemographic characteristic and a measure of social anchorage in terms of social network analysis,^26^ and included whether the respondent had lived out of prison since March 2020 (pre-pandemic) and how long they had been in prison during this stay.

To measure vaccination attitudes and behaviour, respondents were asked whether they had been vaccinated against COVID-19. Respondents who reported vaccination were asked why they received the vaccine, selecting all reasons that applied and their main reason. Respondents who reported not being vaccinated were asked why not, again selecting all reasons that applied and a main reason (Table 1).

**Table 1:** Reasons selected for being vaccinated (left-hand columns) and not being vaccinated (right-hand columns). Second and fifth columns are percentages of respondents selecting a reason when asked to tick as many boxes as apply; third and sixth columns are when asked to select the single main reason.

Reasons vaccinated			Reasons not vaccinated		
Reason	Given as a reason	Given as main reason	Reason	Given as a reason	Given as main reason
‘To protect myself’	83.3%	59.5%	*‘I don’t think I will catch COVID-19*	14.0%	4.1%
‘To avoid spreading it to others’	62.3%	18.1%	*‘I don’t believe in COVID-19’*	27.4%	12.3%
‘To help society return to normal’	43.1%	6.9%	*‘I can’t be bothered’*	12.9%	2.9%
‘To help prison life return to normal’	40.3%	4.4%	*‘I would prefer to protect myself in other ways (avoiding others, wearing a mask etc.)’*	21.1%	1.8%
‘Family told me to get vaccinated’	24.2%	5.8%	*‘I would rather get a vaccine outside prison’*	4.7%	1.2%
‘Friends told me to get vaccinated’	15.9%	1.4%	*‘COVID-19 is not a serious illness’*	14.,6%	2.3%
‘So that I don’t have to keep wearing a mask’	21.2%	3.4%	*‘If I got COVID-19, I would just get treated’*	7.0%	2.3%
‘To help protect the NHS’	43.8%	5.8%	*‘The vaccine might have side effects’*	38.6%	8.2%
‘So I can go to pubs/clubs/travel’	21.0%	3.6%	*‘The vaccine might affect my fertility’*	18.1%	2.3%
Other	11.1%	3.6%	*‘The vaccines don’t work’*	25.1%	7.0%
Don’t know	0.8%	1.0%	*‘I want to wait until there is more evidence about the vaccine’*	29.2%	7.6%
Prefer not to say	0.6%	1.0%	*‘I want to see if others get vaccinated’*	1.8%	0%
			*‘I don’t trust those giving the vaccine’*	16.4%	2.9%
			*‘I don’t believe in vaccines’*	17.0%	3.5%
			*‘I don’t like injections’*	16.4%	3.5%
			*‘I don’t like feeling pressurised to be vaccinated’*	25.7%	4.1%
			*‘Friends or family told me not to’*	9.4%	0.6%
			*‘I would rather develop natural immunity’*	35.7%	12.3%
			*‘I have been too busy’*	2.9%	1.2%
			*‘I don’t know how to get a vaccine’*	1.2%	0%
			*‘I’m not eligible for health reasons’*	1.8%	1.2%
			*‘I have a religious objection to being vaccinated’*	4.1%	1.2%
			*‘I have already had COVID-19’*	18.1%	2.9%
			*‘I feel overwhelmed by the decision’*	5.3%	1.2%
			*‘There is a hidden agenda behind the vaccine campaign’*	19.9%	8.2%
			*‘The vaccines can give you COVID-19’*	11.7%	2.3%
			*‘I need more information’*	9.9%	4.1%
			*‘None of the above’*	5.3%	3.5%
			*Not stated*	1.8%	20.5%

For the social capital and social support variables, we used four groups of items. First, we measured civic and institutional trust (cognitive social capital)^27^ using a series of items with the stem: ‘How much do you trust these groups?’, where respondents rated their level on a 0–10 scale, with 0 labelled as ‘Not at all’ and 10 as ‘Completely’. The groups were as follows: ‘Most people in general’, ‘People you know personally in prison’, ‘People you know personally outside of prison’, ‘UK Government’, ‘Welsh Government’, ‘Your doctor or nurse in prison’, Your doctor or nurse outside of prison’, ‘Prison staff’, ‘The media (e.g. TV, newspapers, radio stations)’, ‘Scientists’, ‘Social media’, and ‘Pharmaceutical companies’. Second, we measured relationships with staff (vertical social capital^16^) and the extent of institutional engagement by asking ‘Who might you go to for support whilst in prison?’, with the response options: ‘other prisoners’, ‘prison officers’, ‘teachers’, ‘psychology’, ‘chaplaincy’, ‘keyworker/offender manager’, ‘substance misuse/drug worker’, ‘healthcare’, ‘listeners’, ‘telephone helplines e.g. Samaritans’, ‘friends or family’, and ‘prefer not to say’; with respondents asked to tick all that applied. Third, we considered bonding social capital (relationships with friends, family, and fellow prisoners) by asking ‘How often do you phone family or friends who are outside prison?’ We also asked ‘How often do you have social visits from friends or family?’

### Analyses

We computed descriptive statistics of the above variables. We fitted binomial generalised linear mixed effects models,^28^ to test individually whether each variable predicted vaccination. Odds ratios with 95% confidence intervals were used to assess the strength of relationships and whether they exceeded what would be expected by chance. Models contained random intercepts for the prison and wing, to account for nesting of data, and fixed effects for each predictor, with categorical variables dummy-coded as appropriate. A separate model was fitted for each predictor to avoid issues with ‘Table 1 fallacy’.^29^

Unplanned multivariate versions of the models for social capital variables, adjusting for age and ethnicity, were fitted to assess whether these associations were driven by confounding.

## Results

### Sample

Surveys were completed by 727 respondents in six prisons. Of these, 496 (68%) reported receiving at least one vaccine, 171 reported being unvaccinated (24%), 34 preferred not to respond, and 26 did not complete the item. Table 2 describes the sociodemographic characteristics of the sample.

**Table 2:** Composition of sample on variables of study (reasons for and against vaccination reported in Table 1, social capital variables reported in Tables 3 and 4).

	*N* (%)
Total sample size	727 (100)
Vaccinated against COVID-19 (%)	
Yes, I've had 1 or more vaccines	496 (68.2)
No	171 (23.5)
Prefer not to say	34 (4.7)
Not stated	26 (3.6)
Age group (%)	
18–20 years	32 (4.4)
21–30 years	187 (25.7)
31–40 years	220 (30.3)
41–50 years	157 (21.6)
51–60 years	71 (9.8)
61–70 years	36 (5.0)
71–80 years	14 (1.9)
81 years or older	7 (1.0)
Not stated	3 (0.4)
Ethnicity (%)	
White	573 (78.8)
Mixed/Multiple ethnic groups	36 (5.0)
Asian/Asian British/Asian Welsh	41 (5.6)
Black/African/Caribbean/Black British/Black Welsh	48 (6.6)
Other ethnic group	12 (1.7)
Prefer not to say	13 (1.8)
Not stated	4 (0.6)
Education (%)	
Other	244 (33.6)
GCSE/A level	111 (15.3)
Do not know	48 (6.6)
Not stated	26 (3.6)
Prefer not to say	75 (10.3)
None	164 (22.6)
University	59 (8.1)
General health (%)	
Very good	219 (30.1)
Good	288 (39.6)
Fair	153 (21.0)
Bad	38 (5.2)
Very bad	13 (1.8)
Prefer not to say	15 (2.1)
Not stated	1 (0.1)
Since March 2020 have you spent any time living outside prison? (%)	
Yes	469 (64.5)
No	237 (32.6)
Not stated	21 (2.9)
For this prison stay, how long have you been in prison? (%)	
0–3 months	88 (12.1)
4–6 months	96 (13.2)
7–12 months	160 (22.0)
1–2 years	147 (20.2)
More than 2 years	222 (30.5)
Not stated	14 (1.9)
Vaccinated against influenza (%)	
Yes	398 (54.7)
No	253 (34.8)
Not sure	36 (5.0)
Prefer not to say	24 (3.3)
Not stated	16 (2.2)
Vaccinated against hepatitis B (%)	
Yes	325 (44.7)
No	245 (33.7)
Not sure	122 (16.8)
Prefer not to say	21 (2.9)
Not stated	14 (1.9)
Previous COVID-19 infection (%)	
Yes ‚ confirmed by test	373 (51.3)
Yes ‚ not confirmed by test	50 (6.9)
No	212 (29.2)
Do not know	63 (8.7)
Prefer not to say	21 (2.9)
Not stated	8 (1.1)
How often do you phone family or friends who are outside prison? (%)	
Daily	386 (53.1)
2–3 times per week	107 (14.7)
Weekly	70 (9.6)
Every 2 weeks	25 (3.4)
Monthly	18 (2.5)
Less than monthly	13 (1.8)
Never	63 (8.7)
Prefer not to say	34 (4.7)
Not stated	11 (1.5)
How often do you have social visits from friends or family? (%)	
Weekly	208 (28.6)
Monthly	201 (27.6)
Every 2–3 months	46 (6.3)
Every 4–6 months	26 (3.6)
Less than every six months	25 (3.4)
Never	164 (22.6)
Prefer not to say	40 (5.5)
Not stated	17 (2.3)

### Characteristics

Vaccination uptake increased with age and was higher in White respondents, although confidence intervals overlapped with one for all groups except the Asian, other, and not stated groups. The level of education did not affect uptake.

Respondents who had been in prison for shorter periods had lower vaccination rates than those who had been in for 1–2 years, with a trend in the same direction for those who had been in for over 2 years. Those who had spent time outside of prison since the start of the pandemic had similar vaccination uptake to those who had not.

The highest vaccination rates were those reporting ‘good’ general health, with lower uptake above and below this.

Those reporting a confirmed previous infection with COVID-19 had higher vaccine uptake than those reporting no previous infection. Reporting being vaccinated against hepatitis-B and influenza was associated with being vaccinated against COVID-19.

### Vaccine attitudes and behaviours

Of those vaccinated, ‘to protect myself’ was the most common reason to accept vaccination (Table 1), but respondents reported other reasons related to protecting others, protecting the NHS, and returning to normal.

Many unvaccinated respondents did not identify the main reason. The reasons given were diverse, likely representing genuine heterogeneity, and the greater range of options was also given.

Respondents gave reasons that suggested COVID-sceptical attitudes, such as ‘I don’t believe in COVID-19’, ‘COVID-19 is not a serious illness’, and ‘There is a hidden agenda behind the vaccine campaign’, with almost 25% giving one of these as the main reason. Over 25% of unvaccinated respondents gave not believing in COVID-19 as a reason for not getting vaccinated.

Many reported concerns about side effects or desire for more certainty about safety: ‘The vaccine might have side effects’, ‘The vaccine might affect my fertility’, ‘I want to wait until there is more evidence about the vaccine’, and ‘I need more information’, with ~22% giving one of these as the main reason. ‘The vaccines don’t work’ and ‘I would rather develop natural immunity’ were also common reasons, suggesting vaccine-sceptical attitudes. Very few cited logistical barriers such as ‘I have been too busy’, ‘I’m not eligible for health reasons’, or ‘I don’t know how to get a vaccine’, and few would rather wait to be vaccinated outside of prison.

### Social capital and support

Nearly all trust measures were positively associated with vaccination (Table 4). Confidence intervals for trust in people known personally in prison (95% CI 0.958–1.081) and in social media (95% CI: 0.987–1.150) overlapped with 1, but otherwise, higher trust was associated with being vaccinated across the board. Adjusting for age and ethnicity made little difference to associations, although confidence intervals overlapped with one for ‘People you know personally outside prison’ and ‘Your doctor or nurse outside of prison’, and became significant for ‘social media’, after adjustment.

The number of sources of support identified in response to the question ‘Who might you go to for support whilst in prison?’ predicted vaccine uptake, as did specific sources of support. Adjusting for age and ethnicity attenuated the association for the number of sources of support. Adjustment had a varied effect on the specific support sources. Most changed little, but ‘prison officers’ became non-significant, and ‘Telephone helplines e.g. Samaritans’ became significant after adjustment.

Contact with friends and family from outside the prison was not associated with vaccination (Table 3).

**Table 3:** Odds ratios for prediction of COVID-19 vaccination by sociodemographic, health-related, prison stay, and contact with friends and family variables.

Variable	Level	Vaccination rate	OR	OR_95% CI_
Age	18–20	59.3%	[Reference]	[Reference]
	21–30	66.5%	1.37	0.592–3.15
	31–40	75.0%	2.07	0.899–4.79
	41–50	79.6%	**2.69**	**1.13–6.41**
	51–60	80.9%	**2.92**	**1.09–7.77**
	61–70	87.9%	**4.98**	**1.36–18.31**
	71–80	85.7%	4.02	0.73–22.07
	81+	33.3%	0.330	.026–4.25
	Not stated	33.3%	0.345	0.280–4.32
Ethnicity	White	77.8%	[Reference]	[Reference]
	Mixed/Multiple ethnic groups	65.6%	0.544	0.253–1.168
	Asian/Asian British/Asian Welsh	61.3%	**0.435**	**0.202–0.936**
	Black/African/Caribbean/Black British/Black Welsh	67.4%	0.592	0.301–1.164
	Other ethnic group	27.3%	**0.1049**	**0.027–0.407**
	Prefer not to say	55.6%	0.3459	0.090–1.330
	Not stated	25.0%	**0.097**	**0.010–0.957**
Education	No formal qualifications	69.5%	0.739	0.466–1.173
	GCSE/A levels	78.8%	1.196	0.682–2.097
	University	78.6%	1.162	0.569–2.372
	Other	75.5%	[Reference]	[Reference]
	Don’t know	81.0%	1.417	0.614–3.271
	Prefer not to answer	67.2%	0.665	0.359–1.232
	Not stated	70.0%	0.769	0.279–2.120
General health	Very good	69.5%	**.567**	**0.370–0.869**
	Good	79.8%	[Reference]	[Reference]
	Fair	74.5%	0.721	0.443–1.175
	Bad	71.4%	0.612	0.273–1.369
	Very bad	44.4%	**0.181**	**0.045–0.722**
	Prefer not to say	66.7%	0.468	0.081–2.694
	Not stated	0.0%	–	–
Previous COVID -19 infection	Yes—confirmed by test	79.8%	[Reference]	[Reference]
	Yes—not confirmed by test	68.1%	0.535	0.273–1.052
	No	67.0%	**0.504**	**0.338–0.751**
	I don’t know	71.7%	0.634	0.328–1.225
	Prefer not to say	57.1%	0.302	0.063–1.439
	Not stated	83.3%	1.242	0.140–11.010
Vaccinated against Influenza	Yes	88.0%	**5.736**	**3.901–8.552**
	No	56.0%	[Reference]	[Reference]
	Prefer not to say	57.9%	1.080	0.423–2.870
Vaccinated against hepatitis-B	Yes	79.4%	**1.622**	**1.133–2.334**
	No	70.3%	[Reference]	[Reference]
	Prefer not to say	65.0%	0.784	0.311–2.139
For this prison stay, how long have you been in prison?	0–3 months	67.9%	[Reference]	[Reference]
	4–6 months	71.3%	1.190	0.612–2.313
	7–12 months	71.2%	1.220	0.671–2.219
	1–2 years	79.1%	**1.892**	**1.004–3.563**
	More than 2 years	77.7%	1.699	0.948–3.048
	Not stated	71.4%	1.188	0.338–4.182
Since March 2020 have you spent any time living outside prison?	Yes	73.3%	[Reference]	[Reference]
	No	76.4%	1.186	0.806–1.743
	Not stated	76.5%	1.205	0.382–3.802
How often do you phone family or friends who are outside prison?	Daily	73.7%	[Reference]	[Reference]
	2–3 times per week	77.9%	1.244	0.736–2.105
	Weekly	76.5%	1.108	0.595–2.064
	Every 2 weeks	78.9%	1.296	0.413–4.061
	Monthly	66.6%	0.694	0.248–1.937
	Less than monthly	76.9%	1.209	0.322–4.546
	Never	74.1%	0.956	0.496–1.843
	Prefer not to say	52.6%	**0.375**	**0.145–0.969**
	Not stated	100%	–	–
How often do you phone family or friends who are outside prison?	Weekly	0.7202073	[Reference]	[Reference]
	Monthly	0.7864583	1.419	0.886–2.274
	Every 2–3 months	0.7045455	0.904	0.435–1.876
	Every 4–6 months	0.7600000	1.214	0.456–3.233
	Less than every six months	0.5909091	0.560	0.223–1.405
	Never	0.7516340	1.101	0.663–1.829
	Prefer not to say	0.6000000	0.559	0.233–1.341
	Not stated	100%	–	–

**Table 4:** Odds ratios for prediction of COVID-19 vaccination by trust and social support source variables.

Group	Variable	Mean/total/percentage for vaccinated	Mean/total/percentage for unvaccinated	OR	OR_95% CI_	Adjusted OR	Adjusted OR_95% CI_
Trust (note: separate models fitted for each variable)	Most people in general	5.746	4.724	**1.146**	**1.072–1.225**	**1.130**	**1.057–1.209**
	People you know personally in prison	6.364	6.234	1.017	0.958–1.081	1.010	0.949–1.075
	People you know personally outside prison	8.033	7.482	**1.063**	**1.003–1.127**	1.051	0.990–1.116
	UK government	3.457	2.788	**1.109**	**1.030–1.194**	**1.129**	**1.047–1.219**
	Welsh Government	3.646	3.043	**1.086**	**1.013–1.164**	**1.099**	**1.022–1.181**
	Your doctor or nurse in prison	5.775	5.030	**1.069**	**1.012–1.130**	**1.063**	**1.004–1.125**
	Your doctor or nurse outside of prison	7.265	6.634	**1.066**	**1.006–1.129**	1.056	0.994–1.122
	Prison staff	5.175	4.263	**1.105**	**1.040–1.175**	**1.083**	**1.018–1.152**
	The media (e.g. tv, newspapers, radio stations)	3.688	3.164	**1.079**	**1.006–1.158**	**1.085**	**1.009–1.166**
	Scientists	5.422	4.534	**1.093**	**1.031–1.158**	**1.085**	**1.022–1.152**
	Social media	3.215	2.848	1.065	0.987–1.150	**1.081**	**1.000–1.170**
	Pharmaceutical companies	4.700	3.584	**1.140**	**1.069–1.216**	**1.136**	**1.064–1.214**
Who might you go to for support whilst in prison? (note: separate models fitted for each variable)	Total count of sources	2.496	1.641	**1.228**	**1.110–1.358**	**1.232**	**1.109–1.367**
	Other prisoners	50.8%	38.0%	**1.567**	**1.080–2.273**	**1.618**	**1.100–2.378**
	Prison officers	39.3%	28.7%	**1.493**	**1.01–2.208**	1.437	0.960–2.152
	Teachers	15.1%	8.8%	1.790	0.983–3.259	1.657	0.895–3.067
	Psychology	10.9%	8.2%	1.292	0.688–1.425	1.409	0.730–2.720
	Chaplaincy	25.0%	14.6%	**1.849**	**1.143–2.991**	**1.853**	**1.122–3.061**
	Key worker/offender manager	24.8%	10.5%	**1.803**	**1.114–2.916**	**1.873**	**1.146–3.061**
	Substance misuse/drug worker	17.1%	8.8%	**2.010**	**1.148–3.840**	**2.247**	**1.208–4.179**
	Healthcare	35.3%	15.8%	**2.810**	**1.773–4.457**	**2.866**	**1.769–4.644**
	Listeners	17.9%	10.5%	**2.029**	**1.139–3.614**	**2.130**	**1.174–3.863**
	Telephone helplines e.g. Samaritans	8.9%	4.7%	1.885	0.859–4.137	**2.457**	**1.053–5.729**
	Friends or family	56.0%	59.6%	0.721	0.488–1.064	0.734	0.489–1.102
	Prefer not to say	5.2%	10.5%	0.458	0.242–0.868	**0.451**	**0.227–0.896**

## Discussion

### Main finding of this study

We surveyed over 15% of the Welsh prison population on COVID-19 vaccination attitudes and behaviour. Respondents were male, younger, more ethnically diverse, held fewer educational qualifications, and reported poorer health than the Welsh population.^30^

We found predictors of vaccine uptake across several domains, including demography (increasing age and White ethnicity), health (self-perceived good health, previous COVID-19 infection, and previous hepatitis-B or influenza vaccination), and social capital (increasing with sources of support within prison and increasing levels of trust in general).

### What is already known on this topic

Rates of having received at least one COVID-19 vaccination were lower (68.2%) than the figure for the Welsh population aged 5+ in July 2022 (86%),^31^ confirming previous evidence that prison populations have lower rates of vaccine uptake than the general community.^9^^,^^10^

The most common reason for accepting COVID-19 vaccination was to ‘protect myself’. This echoes findings in the United States^32^ and Spain.^33^ Other reasons included protecting others and helping life return to normal, although very few gave these as their main reason.

High levels of disbelief in the COVID-19 vaccine have been previously reported amongst prison populations, and our findings support this. In Pakistan, 60% of prisoners did not believe COVID-19 was a real problem and 50% believed the pandemic was a conspiracy.^34^ Over a quarter of our respondents stated they did not believe in COVID-19 with 12% giving this as their main reason against vaccination. Almost a quarter gave their main reason for declining vaccination as either not believing in COVID-19 or not believing in vaccines more generally. Twenty percent of respondents felt there was a hidden agenda behind the vaccine campaign. These results suggest the need to address disbelief in COVID-19 before persuading of vaccination benefits.

Concerns about vaccine side effects have been shown elsewhere to be a common reason for declining vaccination, so it is unsurprising that our results supported this. International literature describes high levels of concern about side effects from prisons in the United States, Italy, Canada, and Pakistan.^32^^,^^34–36^ Just under 40% of our respondents were concerned about side effects, 18% believed the vaccine might affect fertility, and just under 30% wanted to wait until there was more evidence about vaccines. The broad distrust seen in survey responses and wider literature suggests a need to tailor communications to those who are imprisoned, build trust, and involve service users in the design and delivery of health promotional material and messaging. Encouragingly, there is evidence to suggest that this method of increasing education on vaccination improves uptake in prisons.^11^

Reporting being vaccinated against hepatitis-B and influenza was both associated with vaccination against COVID-19, particularly influenza. This is similar to findings in US prison populations where COVID-19 vaccine refusal was strongly associated with not receiving influenza vaccines,^37^ and in Italy where COVID-19 vaccine uptake was higher in those reporting influenza vaccination.^38^ This could suggest that broader attitudes to vaccination help shape attitudes to COVID-19 vaccination but may also be driven by individual health risk status.

### What this study adds

This study extends the evidence on social capital as an important determinant of vaccination to the prison setting, which is an interesting and important context in terms of vaccination policy and from a social capital perspective. Prison residents reporting low trust in people and institutions, in general, were less likely to be vaccinated. This supports findings from previous studies that trust is at the core of social capital and should be promoted to support engagement with health in prison.^21^ Social capital can be a double-edged sword when it comes to health, as risky health behaviours can travel along the same social networks as healthy ones do.^39^ We did not observe evidence of this here, but this may depend on how social capital is measured, as different aspects of social capital have been shown to have different associations with COVID-19 outcomes.^15^^,^^40^

Likewise, respondents reporting fewer sources of social support in prison were less likely to be vaccinated. This echoes findings in community populations^41^^,^^42^ that social isolation is associated with vaccine hesitance, highlighting the importance of ensuring that prison residents are not socially isolated. Reporting staff support through chaplaincy, teachers, prison officers, key workers, substance misuse workers, and listeners suggested increased COVID-19 vaccine uptake. These findings suggest that investing in prison staff support structures may reap secondary benefits for health. Engaging staff to support access to vaccines may also help improve uptake. Building social capital through improved social support structures and trusted relationships will improve the health and wellbeing of people in prison. This can be achieved through evidenced methods such as peer support,^21^ or prison social groups to support health interventions.^22^

Literature^43^ suggests that those in prison may draw on sources of social capital from outside the prison, as well as inside it. Interestingly, the frequency of phone calls and visits from friends and family was not associated with vaccination, nor was social support from friends and family, suggesting that social support from outside prison is not a substitute for sources of support inside.

International literature suggests that some prison cohorts may be reluctant to receive vaccination whilst in custody, having more trust in community healthcare.^10^ Encouragingly, we did not find this, with only 5% of respondents indicating they would rather receive a vaccine outside of prison. Only 1% of respondents stated they did not know how to get the vaccine whilst in prison, suggesting that logistical barriers are not responsible for the vaccination gap within the community population.

### Limitations of this study

The survey was voluntary, and therefore respondents may not be representative of the prison population. More mistrustful residents may have been less likely to respond and less likely to be vaccinated. Survey research in prisons is logistically challenging and compromises were made in questionnaire design and fieldwork—some sensitive topics, such as mental health, were avoided as we could not guarantee confidentiality for respondents. All sites were male prisons; perspectives may differ in female prisons. Results reflect the post-pandemic period in late 2022. Since then, COVID-19 incidence has decreased and COVID-19 booster vaccination in the UK has been limited to those over 65 years of age or at increased clinical risk.^44^ Despite this, prison populations will always be at heightened risk for the spread of respiratory infections, so COVID-19 vaccination will be an important driver of prison health for the foreseeable future. Finally, we measured social capital by asking about sources of social support and trust in various groups. However, prisons represent complex social settings and other forms of social capital that we did not focus on; *e.g.* membership in organised gangs and relationships with cellmates’ may be important. Future work should develop new measures of prison social capital so that we can better understand its relationship with health. Furthermore, we focused on individual measures of social capital, rather than measuring the social environment on, e.g. specific blocks of prisons. Further work could attempt to assess group-level social capital.

## Conclusion

The study supports that social capital is a valuable public health resource. The association between social support and vaccine uptake suggests that policies to improve social support structures for prisoners may pay dividends for health. For infectious diseases, such as COVID-19, these benefits may extend beyond the prisoner receiving interventions if this interrupts transmission chains. Second, the findings may support targeting specific groups within the prison population to improve uptake, namely those who are younger, serving shorter sentences, who consider themselves to be in poorer health, who feel less socially supported, and who feel more distrustful of others. Third, some reasons given for non-vaccination appear amenable to persuasion, so the vaccination gap between the prison and community populations may be able to be narrowed with the right vaccination campaign. Work on specific message framings for prisoners is needed including addressing beliefs about COVID-19 as well as the vaccine. Fourth, vaccination is not enough to prevent prison outbreaks and must be considered alongside other measures to strengthen infection prevention and control.

## Notes

**Stephanie E. Perrett,** Consultant Nurse

**Daniel Rhys Thomas,** Consultant Epidemiologist

**Christopher W. N. Saville,** Clinical Senior Lecturer
